# Spin-dependent manipulating of vector beams by tailoring polarization

**DOI:** 10.1038/srep34276

**Published:** 2016-09-28

**Authors:** Junxiao Zhou, Wenshuai Zhang, Yachao Liu, Yougang Ke, Yuanyuan Liu, Hailu Luo, Shuangchun Wen

**Affiliations:** 1 Laboratory for Spin Photonics, School of Physics and Electronics, Hunan University, Changsha 410082, China

## Abstract

We examine the spin-dependent manipulating of vector beams by tailoring the inhomogeneous polarization. The spin-dependent manipulating is attributed to the spin-dependent phase gradient in vector beams, which can be regarded as the intrinsic feature of inhomogeneous polarization. The desired polarization can be obtained by establishing the relationship between the local orientation of polarization and the local orientation of the optical axis of waveplate. We demonstrate that the spin-dependent manipulating with arbitrary intensity patterns can be achieved by tailoring the inhomogeneous polarization.

Polarization is a fundamental property of light that has received much attention in the light-matter interactions. Recently there has been an increasing interest in light beams with spatially variant states of polarization^1^,^2^,^3^,^4^. Spatially arranging the states of polarization is expected to lead to new effects and phenomena that can expand the functionality and enhance the capability of optical systems^5^,^6^,^7^,^8^,^9^,^10^. The spatially variant polarization states covering the whole Poincaré sphere is called full Poincaré beams, which has been proposed and investigated recently^11^,^12^,^13^. One other special example is laser beams with cylindrical symmetry in polarization, the so-called cylindrical vector beams. This kind of vector beam possesses a uniform polarization rotation rate in azimuthal direction, and can be decomposed into two vortex beams with opposite spin states^14^,^15^,^16^,^17^. Due to the overlying of left- and right-handed components, it is difficult to observe the spin-dependent splitting during the propagation process. However, the spin-dependent splitting can be observed by breaking the rotation symmetry of cylindrical vector beam. This novel kind of spin-dependent splitting occurs in momentum space and can be directly observed in the far field^18^,^19^, which is considered from the point of view in azimuthal phase gradient^20^. However, in all the studies mentioned above, no one steers it in a polarization evolutionary perspective, which is more flexible and powerful to modulate the spin-dependent splitting.

In this work, we propose a general mode to achieve the spin-dependent manipulation of vector beams by tailoring inhomogeneous polarization, which is focused on spin-dependent splitting and extended to spin-dependent focusing. According to the inhomogeneous polarization distribution based on the spin-dependent phase, one can obtain the any desired spin-dependent manipulating. When a fundamental Gaussian beam is modulated by inhomogeneous polarization, the different spin states can be achieved in the far field. For the purpose of verifying this method, we demonstrate the spin-dependent splitting and focusing of the Laguerre-Gaussian (LG) and Hermite-Gaussian (HG) modes. It offers the evidence to support that the spin-dependent manipulating of the vector beam is caused by its intrinsic feature of inhomogeneous polarization.

## Results

### Theoretical analysis

**Figure 1:**
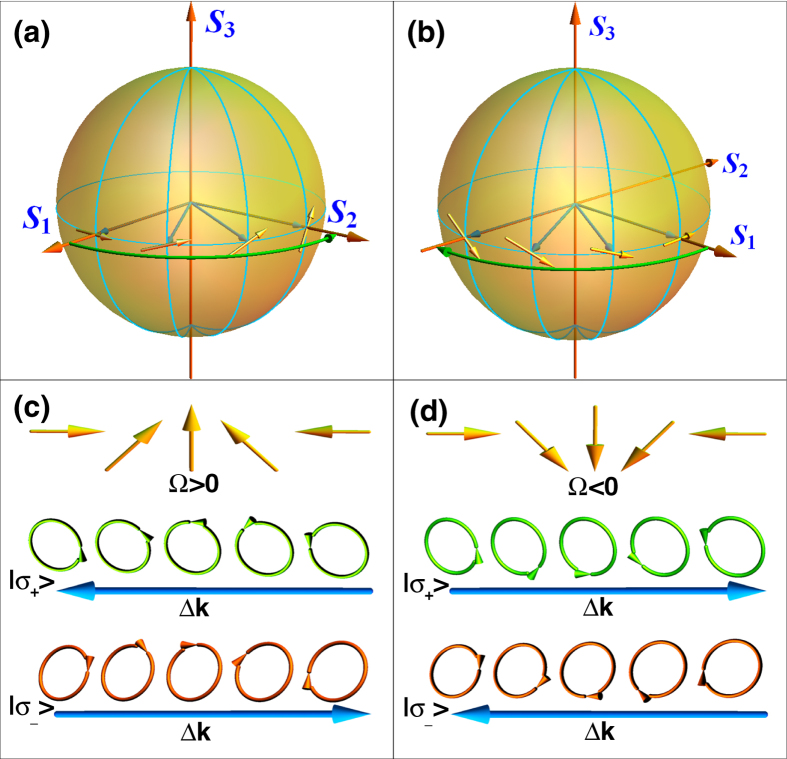
Illustration of the spin-dependent phase in inhomogeneous polarization. The vector beam with a rotation rate Ω > 0 (**a**) and Ω < 0 (**b**) can be unfolded as its local vectorial polarization on the equator of Poincaré sphere. (**c**,**d**) Show the opposite phase gradients of left- and right-spin states in different rotation rates of vector beams.

The polarization of light can be graphically represented by the Poincaré sphere^21^ as shown in Fig. 1(a,b). Figure 1(c,d) show the relationship between the spin-dependent phase and the local orientation of inhomogeneous polarization. The induced spin-dependent phase varies periodically with the rotation of inhomogeneous polarization. The vectorial linear polarization can be decomposed into two spin components with the opposite phase gradients, as depicted in Fig. 1(c,d). It should be note that the varying polarization of Fig. 1(c,d) with a full period can be regard as spanning the equator of the Poincaré sphere in Fig. 1(a,b).

Supposing that a vector beam propagates in *z* direction, and the inhomogeneous polarized light with its electric field can be described by a Jones vector:


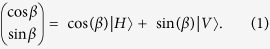


Here, the inhomogeneous polarization can be written as a coordinate-dependent function *β* = Ω*x*, where Ω = *π*/*d* is the polarization rotation rate and *d* represents the period of the polarization variation. For simplicity, we consider the |*H*〉 and |*V*〉 denote the polarization in *x* and *y* directions, respectively. With spin bases |+〉 and |−〉, we have the expression


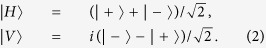


Inserting Eq. (2) into Eq. (1), we have


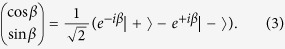


Obviously, the spin-dependent phase can be obtained as Φ = *σ*_±_*β* with *σ*_±_ = ±1 representing the left- and right-handed circular polarizations, respectively. Therefore, the spin-dependent momentum shift is obtained due to the gradient of the geometric phase:


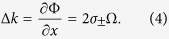


Note that the inhomogeneous polarization indicates a spin-dependent phase gradient, and the spin-dependent momentum shift occurs in *x* direction. The spin-dependent shift with transmitting distance *z* can be described as


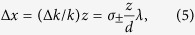


where *k* = 2*π*/*λ* is the wave number of the incident light. The induced shift is related to the period of the polarization variation *d* and the transmitting distance *z* with the wavelength as a constant.

**Figure 2:**
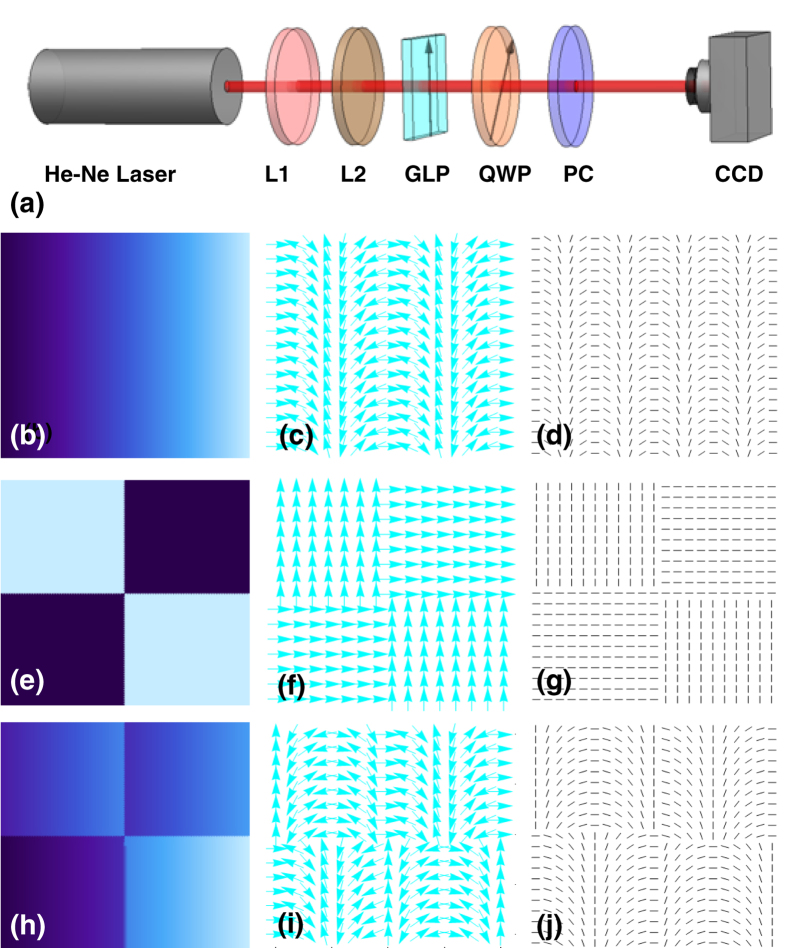
Experimental setup to generate and observe the spin-dependent splitting based on the inhomogeneous polarization. (**a**) A He-Ne laser outputs a linearly polarized Gaussian beam. L stands for lens, GLP represents Glan laser polarizer, QWP is quarter-wave plate, PC represents polarization coWnverter, and CCD denotes charge-coupled device. (**b**–**d**) The relationship among the phase, the polarization and local optical axis orientation of polarization rotation rate in *x* direction. (**e**–**g**) The illustration is the same as those of the first row, but for the case of HG mode. (**h**–**j**) The integrated mode of the HG mode and the polarization rotation rate.

Figure 2(a) shows the experimental setup demonstrates the spin-dependent manipulating of the vector beam based on the inhomogeneous polarization. A He-Ne laser (wavelength *λ* = 632.8 nm) produces a linearly polarized Gaussian beam. A lens system with lens1 (L1) and lens2 (L2) is applied to collimate the Gaussian beam. A Glan laser polarizer (GLP) and a quarter-wave plate (QWP) are employed to generate polarization states we desire. The polarization converter (PC) transforms the local orientation polarization to the local orientation of the optical axis based on the phase feature, which can be regarded as a Pancharatnam-Berry phase optical element^22^,^23^,^24^,^25^, as shown in Fig. 2(b–j). Figure 2(b–d) demonstrate the pattern distributions of polarization rotation rate in *x* direction of the PC, which is used to generate lateral spin-dependent splitting. The distributions of HG mode of PC is employing to implement a single HG beam in second row. The first two rows represent two separation elements. The last row depicts an integrated PC to generate the spin-dependent splitting of HG mode. In fact, the local optical axis orientation of Fig. 2(j) is the sum the superposition of Fig. 2(d,g). The PC was fabricated by femtosecond laser writing of self-assembled nanostructures in silica glass, and the induced form-birefringence patterns can be achieved by manipulating the writing parameters (see also the ‘Methods’ section)^26^. After the laser beam passing though the PC, the output result is recorded by a charge-coupled device (CCD) camera.

Let us consider how PC transforms a linearly polarized field into our desired field. We first obtain a linearly polarized light with the use of GLP and QWP. We assume the linearly polarized input wave with *E*_*x*_ = 1 and *E*_*y*_ = 0, which can be described by a Jones vector


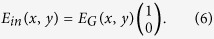


Here, 
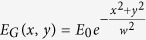
 is a collimated Gaussian beam, *E*_0_ is the amplitude of the field, and *w* is the beam waist. Next, we consider the polarization transformation in the case of a half-wave phase retardation with spatially varying groove orientation. Mathematically, its Jones matrix characterizing the transformation is the expression^27^


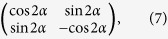


where *α* is the orientation of the fast optical axis. Thus, the field *E*_*out*_(*x*, *y*) behind the PC now takes the form


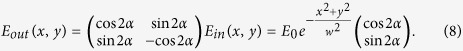


To achieve the spin-dependent splitting with different intensity patterns, it is necessary to impose a phase *φ* describing the intensity pattern of the output beam to the Jones vector in Eq. (3), which has the form


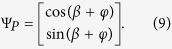


As aforementioned, *β* represents the inhomogeneous polarization with its direction varying in one dimension. Compared with the two Jones matrixes in Eqs (8) and (9), We can clearly take note of the relation *β* + *φ* = 2*α*. Therefore, the PC can theoretically implement any polarization conversions.

We know that the far field diffraction has the property of Fourier transform. Hence, in the paraxial approximation the propagation field behind the PC can be evaluated by Fresnel diffraction formula^28^


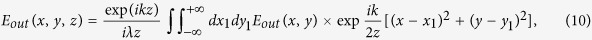


where *x*_1_ (*y*_1_) denotes the dislocation of the splitting from the origin along the *x (y*) axis. The origin of the coordinate is set as the initial position of the incident beam. In classical optics, on account of that the polarization state of light is precisely determined by Stokes vectors *S*_1_, *S*_2_ and *S*_3_ on a Poincaré sphere^29^. Thus, to demonstrate the circular polarization degree of the output electric field and show the separation of spin photons, we consider this common method of detecting the Stokes parameter *S*_3_. The parameter, normalized to the whole intensity in the focal plane, can be given by^30^


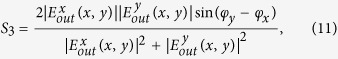


where *φ*_*x*(*y*)_ is the phase of 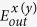 and the denominator is the total intensity in the far-field.

### Result comparison

**Figure 3:**
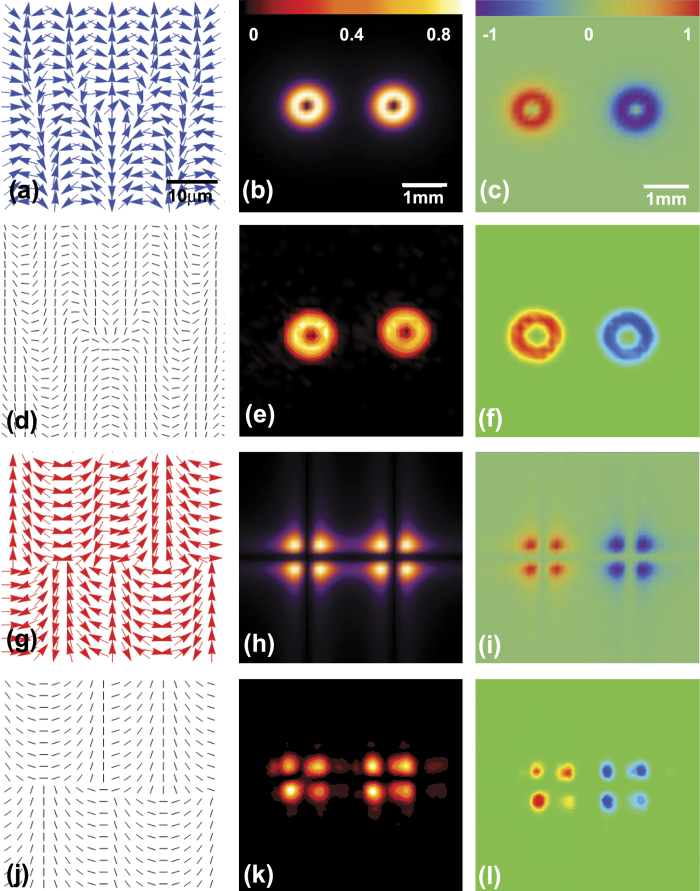
The results of spin-dependent splitting. The first two rows are the theoretical and experimental results of LG mode spin-dependent splitting. The same goes for HG mode in the third and fourth rows. Scale bars, 10 *μ*m, 1 mm and 1 mm in (**a**–**c**). The first column is the distribution of the inhomogeneous polarization (**a**,**g**) and local optical axis orientation (**d**,**j**). The second column presents the intensity distribution. The third column represents normalized Stokes parameter.

We first discuss the spin-dependent splitting of LG and HG modes as illustrated in Fig. 3. The spatial distributions of inhomogeneous polarization, intensity, and normalized Stokes parameter are theoretically plotted in the first and third rows of Fig. 3. Here, *S*_3_ > 0 represents left-handed circular polarization while *S*_3_ < 0 represents right-handed one. In this work, we assume that red and blue denote to the left- and right-handed circular polarizations, respectively. The distribution of inhomogeneous polarization can be decided by the phase. To achieve this spin-dependent splitting, inhomogeneous polarization is consist of two kinds phase, the phase of polarization rotation rate Φ = *σ*_±_*β* and the phase of intensity mode, *φ* of LG or HG [see Eq. (9)]. The expressions of LG and HG modes can be represented as^31^:


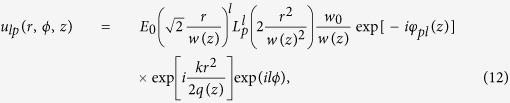



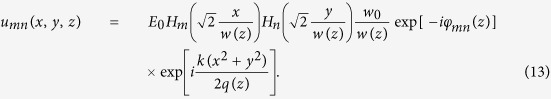


Here, *w*(*z*) represents the beam size, *w*_0_ is the beam size at the beam waist, *H*_*m*_(*x*) is the Hermite polynomials, 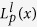 represents the associated Laguerre polynomial, 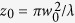 is the Rayleigh length, *q*(*z*) = *z* − *iz*_0_ represents the complex beam parameter, and *φ*_*mn*_(*z*) = (*m* + *n* + 1) tan^−1^(*z*/*z*_0_) and *φ*_*pl*_(*z*) = (2*p* + *l* + 1) tan^−1^(*z*/*z*_0_) are the Gouy phase shifts of HG and LG modes, respectively. For the sake of simplicity and without loss of generality, we choose *m* = 1, *n* = 1 for the HG mode, and *l* = 1, *p* = 0 for the LG mode. Employing the Fresnel diffraction integral with *φ* = Arg(*u*_*lp*_) in the expression Ψ_*P*_, the results are displayed for the spin-dependent splitting of LG mode at the distance of 0.1*z*_0_ in Fig. 3(b,c). This configuration is also used to obtain the intensity and normalized Stokes parameter distribution with *φ* = Arg(*u*_*mn*_) in Fig. 3(h,i), which illustrate consequences of the spin-dependent splitting of HG mode. It evidently resembles the case of LG mode. It is worth mentioning that the spin-dependent splitting of LG and HG modes have been reported in our past work considering a incident LG or HG beam passes through a all-dielectric metasurface^32^. However, it is inherently different from this work, in which the incident beam is a Gaussian mode.

Based on the theoretical results, we design the PC. The distribution of local optical axis orientation of PC is demonstrated in Fig. 3(d), which is employed to achieve the spin-dependent splitting of LG mode. We experimentally obtain the corresponding intensity and normalized Stokes parameter at the same propagation distance, as is shown in Fig. 3(e,f). Similarly, the spin-dependent splitting of HG mode can be achieved in the last row of Fig. 3. The results of Fig. 3(k,l) can be also obtained by two PC with the distribution of local optical axis orientation as shown in Fig. 2(d,g). We could see that the results from the experiment agree well with the theoretical calculation. It is notable that multiple spin-dependent wavefronts carrying different orbital angular momenta is realized by a geometric phase metasurface^33^, which is also an effect method to achieve the spin-dependent modulating as well.

Next, we extend to our scheme for performing the spin-dependent focusing of LG and HG modes. We should reconstruction the inhomogeneous polarization and design the PC, whose local optical axes periodically varies in radial direction. According to the earlier report^34^, we employ the focusing phase and reconstruct the distribution of inhomogeneous polarization Ψ_*P*_ with 
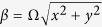
, Ω is the same expression as shown before and Ω = *π*/*d*. Based on Eq. (5), the wavefront is modified and the produced spatial shift becomes


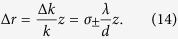


**Figure 4:**
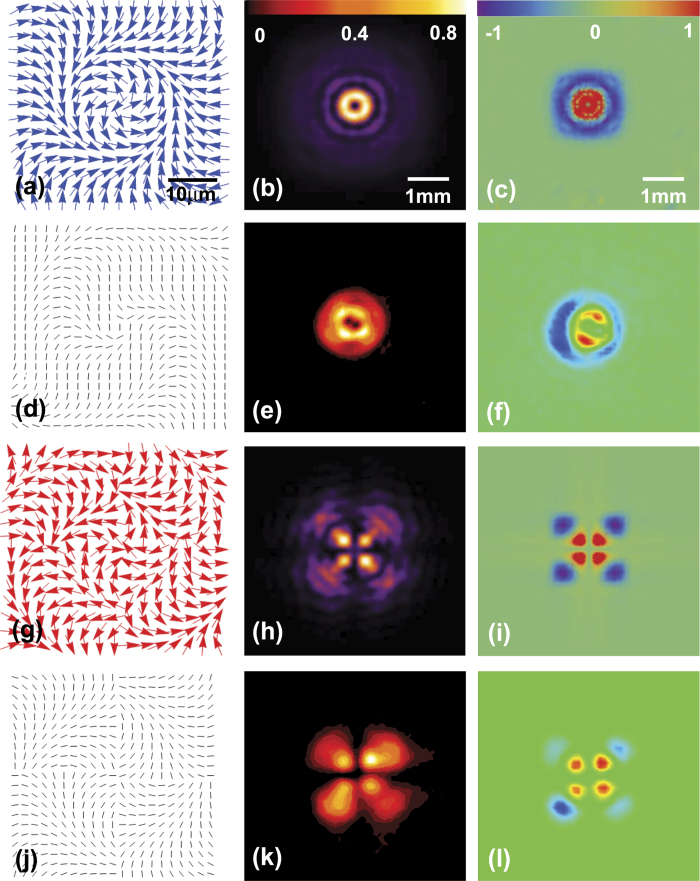
The results of spin-dependent focusing. The first and second rows are the theoretical and experimental results of LG spin-dependent focusing, respectively. The same goes for HG in the third and fourth rows. Scale bars, 10 *μ*m, 1 mm, and 1 mm in (**a**–**c**). The first column is the distribution of the inhomogeneous polarization and local optical axis orientation. The second column presents the intensity distribution. The third column indicates normalized Stokes parameter.

This relationship explains very well that the incident linearly polarized light can be realized the property of spin-dependent focusing, and Δ*r* is a radial displacement. For parameter *φ*, it is the same situation as depicted in Fig. 3. The first row is assumed that *φ* = Arg(*u*_*lp*_), the distribution of the inhomogeneous polarization is calculated in Fig. 4(a). The distribution of intensity and normalized Stokes parameter are theoretically provided by Eqs (10) and (11), respectively, as depicted in Fig. 4(b,c). In our work, we also theoretically exhibit the spin-dependent focusing of HG mode, as shown in third rows of Fig. 4. Here, we choose *φ* = Arg(*u*_*mn*_) in this case. The results in the first and third rows are also displayed at the distance of 0.1*z*_0_.

Likewise, we next experimentally validate the theoretical results by employing the PC. Firstly, we design the optical axis distribution of the PC by the relation of *α* and (*β* + *φ*). According to the result of Fig. 4(a), axis distribution of the spin-dependent focusing of the LG mode can be obtained in Fig. 4(d). The intensity and normalized Stokes parameter are recorded in Fig. 4(e,f). By manipulating the inhomogeneous polarization *φ* = Arg(*u*_*mn*_) and gaining the corresponding optical axis distribution, the spin-dependent focusing of HG mode has also been realized as shown in the last row of Fig. 4. This results of Fig. 4 can be implemented by two independent PC. The experiment results coincide well with the theoretical calculation.

**Figure 5:**
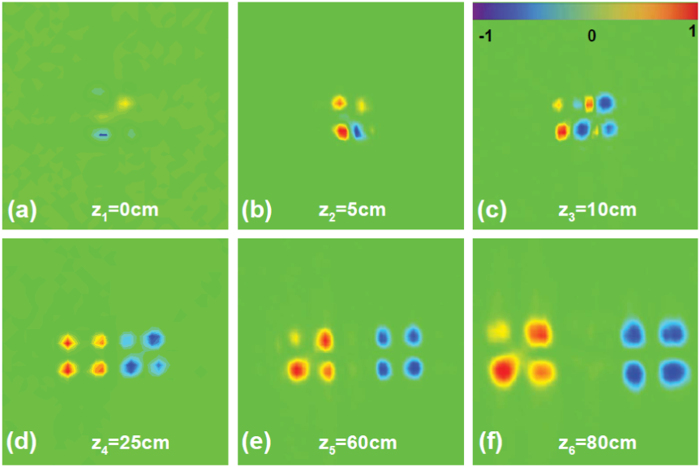
Experimental pattern generated normalized Stokes parameter characteristic of spin-dependent splitting of HG mode is illuminated with different propagation distances.

As we emphasized before, the spin-dependent phase will come into a shift increasing linearly in momentum space with the transmission distance *z*, so the propagation of the vector beam is not stable. Therefore, we consider measuring the instable propagation behavior of the vector beam. We verify the section normalized Stokes parameter of HG mode spin-dependent splitting during propagation for example. The experimental results are exhibited in Fig. 5. As *z* increases, the HG mode gradually splits into two HG beams. Here, all cases are tested under the condition of the period of the polarization variation *d* = 0.25 mm. For *z*_1_ = 0, the spin-dependent splitting does not occur, therefore, the normalized Stokes parameter theoretically should be null, as displayed in Fig. 5(a). For *z*_2_ = 5 cm away from the PC, the separation of the left- and right-handed circular components is still small. At a different distance of *z*_3_ = 10 cm, the normalized Stokes parameter forms a sharp image on the CCD camera. On the contrary, as for the second row, HG mode is completely split, although some parts are slightly blurred. This is mainly because the rotation of the GLP leads to the non-uniformity intensity distribution in the experiment. The result of Fig. 5 also commendably validates the occurrence of the spin-dependent splitting modulated by the PC.

## Conclusion

In conclusion, we have realized the spin-dependent manipulating of vector beams by tailoring polarization. We have demonstrated that the spin-dependent manipulating is attributed to the spin-dependent phase gradient in vector beams, which can be regarded as the intrinsic feature of inhomogeneous polarization. Our experimental results coincide well with the theoretical prediction. These findings suggest that any spin-dependent patterns can be realized by engineering the inhomogeneous polarization. The scheme provides a possible route for the manipulation of spin states of photons, and enables spin-photonics applications.

## Method

The polarization converter is form-birefringent nanostructured glass slabs fabricated by R&D, which is fabricated by using the femtosecond laser at normal incident writing of spatially varying nanogrooves in a silica glass substrate. The glass substrate is placed in a linear air-bearing translation stage system whose rotation speed is controlled by a computer. The substrate of the glass has a diameter of 25 mm, a thickness of 3 mm, and the structured area of 3 mm * 3 mm region centred on the glass substrate. In the case of intense laser irradiation, the uniform glass (SiO_2_) decomposes into porous glass (SiO_2(1−x)_ + xO_2_) whose refractive index is related to the laser intensity. The writing pattern is in subwavelength scale and creates a uniform birefringent phase retardation, which is *ϑ* = 2*π*(*n*_*e*_ − *n*_*o*_)*h*/*λ*. Here *h* is the writing depth and *n*_*e*_ − *n*_*o*_ is the induced birefringence. The effective ordinary and extraordinary refractive indices is expressed as below: 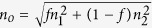, 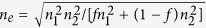. Here, *f* represents the filling factor, *n*_1_ and *n*_2_ denote the refractive indices of the two media which form nanostructured glass slabs. At 633 nm wavelength, the phase retardation of the sample equals *π* with the writing depth being approximately 80 *μm*, the line width is about 30–50 nm and the filling factor is in the range of 0.1–0.2. The transmission efficiency approximately of 60% the polarization converter, and a diffraction efficiency of 80% are measured by a laser power meter at the wavelength of 632.8 nm.

## Additional Information

**How to cite this article**: Zhou, J. *et al.* Spin-dependent manipulating of vector beams by tailoring polarization. *Sci. Rep.* **6**, 34276; doi: 10.1038/srep34276 (2016).
